# Self‐Templated Synthesis of Ultrathin Nanosheets Constructed TiO_2_ Hollow Spheres with High Electrochemical Properties

**DOI:** 10.1002/advs.201600162

**Published:** 2016-07-12

**Authors:** Huiqi Xie, Linfeng Hu, Feilong Wu, Min Chen, Limin Wu

**Affiliations:** ^1^ Department of Materials Science and State Key Laboratory of Molecular Engineering of PolymersFudan UniversityShanghai200433China

**Keywords:** cycle stability, hybrid hollow spheres, Li‐ion battery, specific capacitance, titania, ultrathin nanosheets

## Abstract

TiO_2_ is well‐known nanomaterials and mostly used as solid nanoparticles, and normal hollow spheres for photocatalysts or electrode materials. In this study, a novel self‐templated method is presented to successfully fabricate high‐surface‐area ultrathin nanosheets constructed TiO_2_ hollow spheres through the solvothermal treatment of the titanate–silicone composite particles combined with calcination. The uniquely structured hollow spheres exhibit excellent rate capability and good cycle stability even at a high current density of ≈10 C for the anode material of Li‐ion battery.

## 1 Introduction

Hollow hierarchical nanostructures with properly engineered building blocks have been devoted considerable interest for the fundamental research and practical applications in various fields, such as catalysis, biomedicine, water purification, energy storage and conversion, etc.^1^, ^2^, ^3^, ^4^, ^5^, ^6^, ^7^, ^8^, ^9^

Generally, two main strategies, e.g., template‐based (hard‐ and soft‐templates) and template‐free (Kirkendall effect, galvanic replacement, and Ostwald ripening), are used to obtain well‐defined hollow spheres with different compositions and shapes.^5^, ^10^, ^11^, ^12^, ^13^, ^14^, ^15^, ^16^, ^17^ Recent researches have demonstrated that hollow spheres constructed with 1D nanorods/nanoneedles^18^ and 2D nanosheets^19^, ^20^, ^21^, ^22^ even exhibit faster surface reaction rates and better physicochemical stability because they occupy much larger specific surface areas than the most hollow spheres composed of 0D nanoparticles,^23^ and simultaneously inherit the characteristics of the constituent units. However, synthesis of this kind of hierarchical hollow structures is generally challenging due to the difference of stability between core and shell materials.^24^, ^25^ For example, TiO_2_ is a kind of well‐known nanomaterials as photocatalysts,^26^, ^27^ and anode material of Li‐ion batteries,^28^, ^29^, ^30^, ^31^, ^32^, ^33^, ^34^, ^35^, ^36^, ^37^, ^38^, ^39^, ^40^, ^41^ owing to its low cost, environmental benignity, physicochemical stability, and safety, but most of them are nanoparticles or primary nanoparticle‐constructed hollow spheres, very few researches involve the synthesis of 1D‐ or 2D‐constructed hollow spheres, through template methods.^42^, ^43^, ^44^

In this study, we report a self‐templated synthesis of ultrathin nanosheets constructed titania hierarchical hollow spheres (UNTHS) by hydrothermally treating the titania–silicone composite particles and then calcination. Because the nanosheets favor the short Li^+^ ion diffusion pathway and fast electrochemical reactions, and the hierarchical hollow architectures can still accommodate the volume change and avoid stacking of nanosheets during cycling process, the uniquely structured hollow spheres as the anode material of Li‐ion battery exhibit excellent rate capability and very good cycle stability even at current density of ≈10 C.

## 2 Results and Discussion

### 2.1 Synthesis of Hierarchical TiO_2_ Hollow Spheres

**Figure 1:**
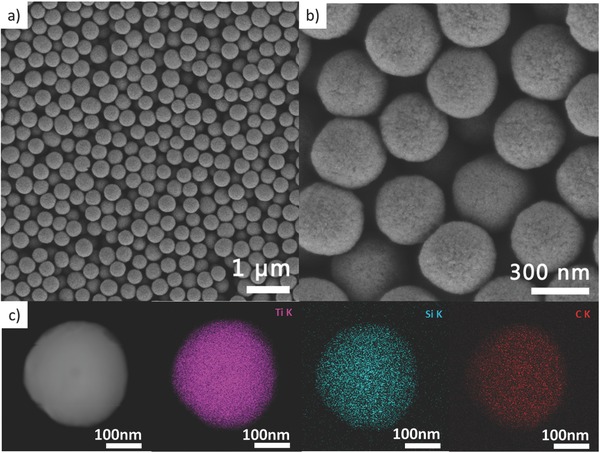
a,b) FESEM images of monodispersed titanate–silicone composite particles. c) EDS images of the single composite gel particle.

The synthesis of UNTHS was started from the pre‐synthesized hybrid particles via the sol‐gel reactions of titania precursor, titanium tetraisopropoxide (Ti(OiPr)_4_), and a small amount of long hydrophobic alkyl silane hexadecylsilanetriols (C_16_TS) as a hydrolysis inhibitor and nucleation agents,^45^ in ethanol under the catalysis of concentrated ammonia. Without C_16_TS, only irregular and nonuniform particles were produced (Figure S1, Supporting Information). The composite particles display monodisperse and spherical morphology with a mean diameter of 380 nm (**Figure** **1**a). The higher magnification SEM image (Figure 1b) indicates that the composite particles with rough surfaces are composed of numerous primary particles. The energy‐dispersive X‐ray spectrometry (EDS) further reveals that 35.2 wt% of Ti and 3.30 wt% of Si are uniformly dispersed in the particles as shown in Figure 1c and Table S1 of the Supporting Information.

**Figure 2:**
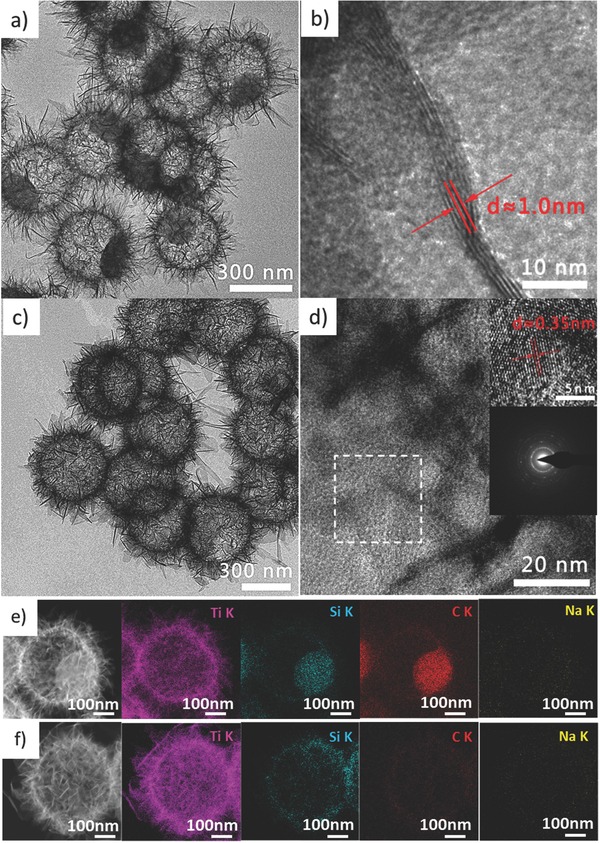
TEM images of a,b) yolk–shell spheres, and c,d) UNTHS. Insets in images d): top, HRTEM image of the retangle aera; bottom, the corresponding SAED pattern. EDS mapping images of e) yolk–shell spheres, and f) UNTHS.

After hydrothermally treated in NaOH solution followed by the H^+^‐exchange process, these titanate–silicone composite particles were successfully converted to yolk–shell composite spheres as shown in **Figure** **2**a. Their X‐ray diffraction (XRD) pattern shows broad diffraction peaks with low intensity (Figure S2a, Supporting Information), which can be indexed to H_2_Ti_5_O_11_·3H_2_O,^46^ suggesting its poor crystallization. Meanwhile, an obvious low angle diffraction peak at around 8.7° indicating the layered structures.^46^, ^47^ The peak marked by black rhombus at around 21.3° could be attributed to polycondensed C_16_TS, like some other organosilica,^48^ which is absent after calcination. Raman spectra show typical titanate phase with weak and broad peaks (Figure S2b, Supporting Information), further confirming the poor crystallized titanate phase.^49^ The yolk is mainly composed of Si and C elements indicated by the EDS mapping images in Figure 2e. And the neglectable amount of Na within the samples reveals that it has been almost entirely removed by the H^+^‐exchange. The inner diameter of the yolk–shell structure is ≈380 nm. The nanosheets constructing the hierarchical microspheres are semitransparent with a thickness of ≈3 nm, and a lamellar structure with a spacing of ≈1.0 nm is observed in high‐resolution transmission electron microscopy (HRTEM) image (Figure 2b), which is corresponding to the XRD results. After calcination at 350 °C, the yolk has been burned away to form a complete ultrathin nanosheets constructed hollow spheres (Figures 2c,f, Supporting Information), and titanate has been transformed into anatase titania with a small content of TiO_2_‐B, which can be confirmed by the XRD (Figure S2a, Supporting Information), and the Raman spectrum (Figure S2b, Supporting Information).^50^ Meanwhile, no obvious peaks of silica are observed due to its small content and amorphous phase, and the low‐angle diffraction peak disappears and a broad peak at ≈12° appears due to the collapse of the interlayer spacing by dehydration and recrystallization.^51^, ^52^ The obtained hierarchical UNTHS still have good morphology, whose Brunauer–Emmett–Teller (BET) surface area and total pore volume are calculated to be ≈285.3 m^2^ g^−1^ and ≈0.88 cm^3^ g^−1^, respectively, based on the N_2_ sorption isotherms (Figure S3, Supporting Information). The thermogravimetric analysis indicates a main weight loss of ≈19.7% (Figure S4a, Supporting Information), which is consistent with the decomposition of titanate to form TiO_2_ and organic components. The HRTEM images (Figure 2d, inset) show nanocrystals within nanosheets with a interspacing of ≈0.35 nm corresponding to the (101) planes of anatase TiO_2_. The selected area electron diffraction (SAED) pattern of the same region reveals well defined diffraction pattern, which can also be assigned to the anatase TiO_2_.^31^ The EDS mapping images of the UNTHS further display that the Ti is absolutely located in the nanosheet‐based shell, only residual silica is dispersed in the inner TiO_2_ shell (Figure 2f).

**Figure 3:**
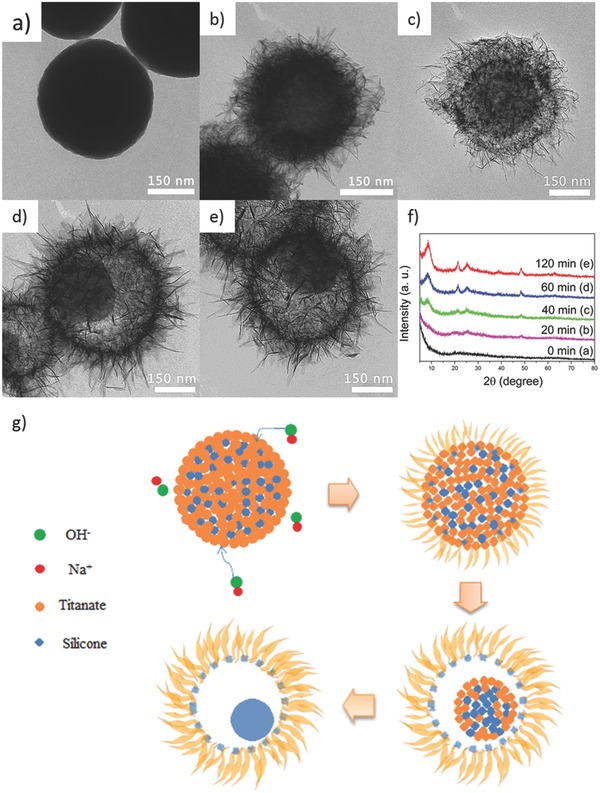
TEM images of the microspheres with hydrothermal treatment at 150 °C for different time: a) 0, b) 20, c) 40, d) 60, e) 120 min. f) The corresponding XRD patterns. g) Scheme of the forming process of the hierarchical titanate yolk–shell spheres.

To illustrate the formation mechanism of the yolk–shell spheres and UNTHS, a time‐dependent hydrothermal treatment experiment in NaOH aqueous solution (1 m) at 150 °C was performed. Products were collected at different stages of the heating process, and their morphologies were subjected to TEM investigation. As shown in **Figure** **3**, only after hydrothermal treatment for 20 min under NaOH solution, some irregular and whisker‐like nanosheets can be seen from the smooth surfaces of the composite particles (Figures 3b vs 3a), forming pompons‐like particles with porous outer layer. The XRD pattern does not show any broad diffraction peak which can be ascribed to amorphous titanate–silicone (Figure 3f), except for only several weak and broad peaks.^25^, ^53^ After continuous hydrothermal etching for 40 min, the core of the pompons‐like particle is shrinking, producing yolk–shell nanostructures with a portion of titanate compositions in the yolk as shown in Figure 3c and Figure S5 of the Supporting Information. Simultaneously, the nanosheets are growing up and an obvious peak at ≈8.5° appears, indicating the formation of lamellar structure. When the reaction time is increased to 60 min, the broad and weak peak at 21.3° become much clearer and sharper, indicating an increasing degree of polycondensation of C_16_TS. And the size of the yolk is further decreased with well defined hierarchical shell (Figure 3d). After 2 h reaction time, the hierarchical yolk–shell structure was kept unchanged (Figure 3e). Meanwhile, the intensity of the low‐angle diffraction peak is obviously enhanced as shown in Figure 3f, suggesting a more pronounced layered structure of the titanate nanosheets.

Based on the above experiments and discussion, the hierarchical microspheres could be formed through an inward etching and outward recrystallization process in a short time scale as shown by the cartoons in Figure 3. With the concentrated NaOH solution is used to assist etching, titanate nanosheets form on the surfaces of the composite particles owing to heterogeneous nucleation, which is energetically favored relative to the homogeneous growth in solution.^54^ And the outer surface is becoming porous, which facilitates the alkali solution penetrating inward for further etching. Meanwhile, the long hydrophobic alkyl C_16_TS molecules distributing uniformly in the composite particles mostly shrink toward core and gradually separate from titanate‐rich phase due to their polycondensation with hydrophobic effect. As a result, the dissolution rate of the amorphous composite particles is gone well with the continuously outward epitaxial growth of titanate nanosheets around the surfaces of the particles to form cavity, which is similar to the Ostwald‐ripening process.^42^, ^55^ And the polycondensed C_16_TS molecules form yolk. The whole etching and recrystallization process come to an end within 60 min, showing high efficiency of the hydrothermal reaction. When these yolk–shell spheres were calcinated, the TiO_2_ hollow spheres composed of ultrathin titania nanosheets were obtained as discussed above.

### 2.2 Electrochemical Properties of the Hierarchical TiO_2_ Hollow Spheres

Considering this unique hollow structure constructed with thin nanosheets, we further investigated their charge storage performance in nonaqueous electrolyte including Li^+^ ions in half‐cells using Li metal as counter and reference electrodes. The principal electrochemical Li^+^ insertion/extraction reaction in a TiO_2_/Li half‐cell can be described as

(1) $${\mathrm{TiO}}_{2}+x{\mathrm{Li}}^{+}+xe^{-}↔{\mathrm{Li}}_{x}{\mathrm{TiO}}_{2}$$

where the coefficient *x* is the amount of inserted Li^+^ in TiO_2_ and the maximum value of it is ≈0.5, which leads to a theoretical storage capacity of 167.5 mAh g^−1^.^56^

**Figure 4:**
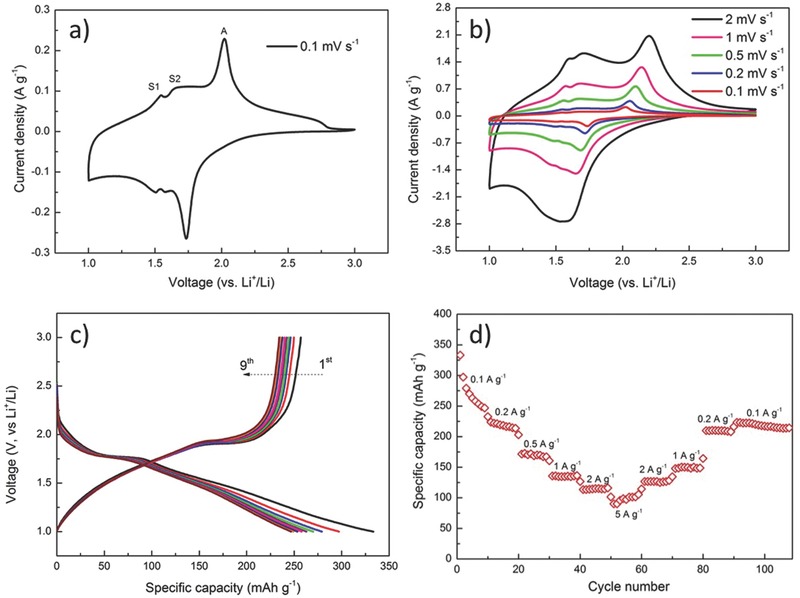
Electrochemical performance of the UNTHS/Li half‐cells. a) CV cuves at scan rate of 0.1 mV s^−1^. b) CV curvers at different scan rates from 0.1 to 2 mV s^−1^. c) Galvanostatic charge and discharge profiles at a current density of 0.1 A g^−1^. d) Rate performance of single UNTHS electrode at different scanning rates of 0.1 to 5 A g^−1^.

As shown in **Figure** **4**a, at a sweep rate of 0.1 mV s^−1^ and in the potential range between 1.0 and 3.0 V (vs Li/Li^+^), the dominated pairs of redox peaks at ≈1.7 and 2.0 V denoted as A of the cyclic voltammetry (CV) are corresponding to the diffusion controlled process of Li^+^ ion into anatase TiO_2_,^57^ while the two minor peaks at around 1.54 to 1.68 V denoted as S1 and S2 are ascribed to the surface Faradaic pseudocapacitive reactions of TiO_2_‐B.^58^, ^59^ Figure 4b shows the CV curves of UNTHS based electrodes obtained at different scan rates ranging from 0.1 to 2 mV s^−1^. As the scan rate increases, all redox peaks are becoming broader and broader, and the peaks S1 and S2 are becoming larger and larger, namely increasing currents as the rate of surface confined faradaic reaction is faster than the diffusion controlled process, indicating that faradaic pseudocapacitance is gradually dominating the electrochemical capacitance at high scan rates, which is favor for high rate capacitance.^60^, ^61^

The galvanostatic charge–discharge voltage scans at current density of 0.1 A g^−1^ over a voltage range of 1.0–3.0 V clearly exhibit three distinct domains (Figure 4c). The first domain is a monotonous voltage drop from 3.0 to ≈1.7 V induced by Li^+^ ion diffusion into the bulk, providing a limit of capacity. The second domain is a characteristic plateau around 1.7 V showing reversible insertion of Li^+^ ion into the anatase TiO_2_, the final domain is a slow decay of the voltage associated with intercalation pseudocapacitance and surface faradaic reaction.^60^, ^62^ At a current rate of 0.1 A g^−1^ (Figure 4c), the discharge capacity at the first cycle is 333.3 mAh g^−1^, while the first charge process releases only 249.8 mAh g^−1^, corresponding to a initial 25.1% capacity loss. From the TGA analysis of the UNTHS (Figure S4b, Supporting Information), the sample calcined during temperature region below 100 °C shows ≈3.4 wt% weight loss which could be attributed to the evorporation of adsorbed moisture due to this highly porous structure, and ≈1.3 wt% weight loss as calcined to 450 °C indicating small amount of carbonaceous residues within the UNTHS structure, which is also found in the EDS results (Figure 2f). These residues and water may cause irreversible reaction of Li^+^ ion and electrolyte decomposition.^63^ It should be noted that a small amount of silica within the UNTHS (12.5 wt% determined from the elemental analysis by inductively coupled plasma‐atomic emission spectrometry (ICP‐AES)) devotes negligible capacity at voltage range of 1.0–3.0 V.^64^, ^65^, ^66^ However, after further cycles, the coulombic efficiency gradually increases to almost 100%. And a reversible capacity of 232.7 mAh ^−1^ during the initial 10 cycles, corresponding to *x* = 0.64 (higher than the theoretical value of 0.5), furthur suggests abundant surface area for Faradaic pseudocapacitive reactions owing to the hierarchical hollow structure.^21^, ^60^

**Figure 5:**
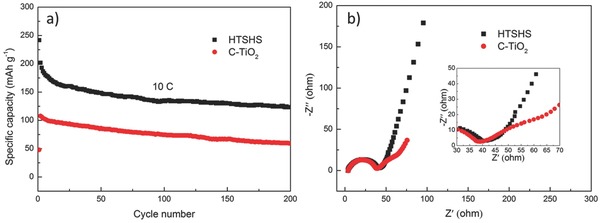
a) Cycle performance of the UNTHS‐ and C‐TiO_2_‐eletrodes at a current density of 2 A g^−1^ (≈10 C); b) Nyquist plots of the UNTHS and C‐TiO_2_.

Figure 4d also displays the rate performance of UNTHS at discharge rates from 0.1 up to 5 A g^−1^, then back to 0.1 A g^−1^, each step lasts for 10 cycles. The UNTHS exhibits high specific capacity of ≈105 mAh g^−1^ even at 5 A g^−1^, indicating that this material with ultrathin titania nanosheets assembled hollow structure has excellent rate capability. Moreover, when the current density returns to 0.1 A g^−1^, the capacity reverts to values above 210 mA h g^−1^ in spite of some capacity decay compared to the first ten cycles, which may be due to the volume swing of the TiO_2_ crystal structure.^19^, ^22^ The UNTHS also demonstrates nice cycling performane. As shown in **Figure** **5**a, despite initial capacity decay, the UNTHS could still deliver discharge capacity as high as ≈123 mAh g^−1^ at a current density of 2 A g^−1^ (≈10 C) after 200 cycles, which is almost double that of the commercial TiO_2_ (C‐TiO_2_, 5–10 nm). As far as we know, these electrochemical properties of the UNTHS‐based anode are better than those of most of the previously reported TiO_2_‐ based electrodes (Table S2, Supporting Information). To understand the improved electrochemical performance of the UNTHS, both the Nyquist plots of UNTHS and C‐TiO_2_ nanocrystals electrodes are shown in Figure 5b, which exhibit depressed semicircle during high‐ and midium‐frequency region, and a straight line in the low‐frequency rigion. The high frequency semicircle is associated with the internal resistance including the interface resistance, the separator, and the charge transfer resistance. The medium‐frequency linear region which are relevant to Li^+^ ion interfacial diffusion resitance, coupled with a double layer capacitance at the interface. It can be seen that the UNTHS is provided with lower ionic resistance and enhanced kinetics of electrolyte peneatration than the C‐TiO_2_. This could be ascribed to the special hierarchical hollow structure assembled with titania nanosheets. The unique hollow titania spheres provide sustainable electrode/electrolyte contact area and reduce diffusion pathways of Li^+^ ions, promoting the electrochemial reactions within the surface to achieve high specific capacitane and good rate performance. Furthermore, the TEM images (Figure S6, Supporting Information) clearly reveal that the UNTHS still maintains the original hierarchical hollow spheres with nanosheets subunits, although the detailed nanosheet structures have somewhat been diminished. Intelligibly, such ultrathin nanosheets are not robust enough to withstand the fast Li^+^ insertion/extraction process under the extended high rate cycling process. The XRD analysis of the UNTHS electrode slurry before and after 200 cycles (Figure S7, Supporting Information) suggested that the anatase crytal structure can still be maintained with no apparent intensity change of the dominate (101) peak. This observation indicates that the hollow structure with interweaving ultrathin nanosheets could efficiently keep them stable rather than aggregation, leading to outstanding cycling stability.

## 3 Conclusion

We have demonstrated the simple and novel synthesis of the ultrathin nanosheets constructed titania hollow spheres through a solvothermal treatment of the titanate–silicone composite particles combined with calcination. When such unique hierarchical TiO_2_ hollow spheres with a high specific area used as the anode of the Li‐ion battery, they can effectively shorten the diffusion path of Li^+^ ions and enlarge contact surface area of electrodes/electrolyte, promoting the electrochemical ractions. Accordingly, they show very good cycle stability even at current density of ≈10 C and excellent rate capacilities.

## 4 Experimental Section

*Synthesis of Composite Titanate–Silicone Paticles*: Ti(O^i^Pr)_4_ (1.42 g, ≈5.0 mmol) was dissolved in hexane (7.0 mL) and heated to 60 °C, and added by C_16_TS (0.25 g, ≈1.0 mmol) in 2.5 mL THF under stirring. This mixture was held at that temperature for 1 h, during which the solvent and resultant isopropyl alcohol were fractionated off continuously under vaccum. When no isopropyl alcohol came out, the composite precursors were obtained and cooled down and kept sealed.

The composite precursors (0.5 g) were dissolved in hexane (1.89 mL, 40 wt%), then added into a flask charged with absolute ethanol (50 mL) and concentrated ammonia solution (0.782 mL, 28 wt%), and the reaction was allowed to proceed at 60 °C for 24 h under continuously vigrous stirring. The product was centrifuged in 5000 r min^−1^ and washed with ethanol for three times, redispersed in deionized water for use.

*Fabrication of Hierarchical TiO_2_ Hollow Spheres*: The dispersion of the above abtained composite paticles (10 mL) was mixed uniformly with NaOH solution (10 mL, 2 m), and then added into a Teflon‐lined stainless‐steel autoclave (30 mL in capacity), and heated to 150 °C for 2 h, and then allowed to cool down to room temperature naturally. The product was washed with HCl aqueous solution (0.1 m) for three times until pH value being close to 7, and immersed in HCl for 2 h, then washed with deionized water and ethanol for several times, and dried at 60 °C in vacuum over night. The obtained products were finally annealed at 350 °C for 6 h at a heating rate of 2 °C min^−1^.

*Characterization*: The surface morphology of the as‐prepared samples was examined by field‐emission scanning electron microscopy (FESEM; Zeiss, Ultra 55, 3 kV), and their microstructures was observed by TEM (FEI, Tecnai G2 20 TWIN, 200 kV) and HRTEM (JEOL JEM‐2010). Crytallographic information was collected using powder XRD (Bruker, D8 Advance X‐ray diffractometer, Cu ΚR radiation (λ = 1.5406 Å)). The Raman spectra were measured on a micro‐Raman spectroscope (Horiba Jobin Yvon, XploRA, the excitation wavelength of 532 nm). The elemental compositions of the samples were analyzed by EDS attached to the FESEM instrument and inductively coupled plasma‐atomic emission spectrometry (ICP‐AES; Hitachi, P4010). The surface area of the samples was measured using a Quantachrome Instruments Quadrasorb evo instrument. Thermogravimetric analysis (TGA) was carried out under a flow of air with a temperature ramp of 10 °C min^−1^.

*Electrochemical Properties*: The electrodes for half‐cell tests were prepared by homogeneously mixing the active materials (e.g., the UNTHS, 70 wt%) with super‐P carbon (20 wt%) and polyvinylidene fluoride (10 wt%) in N‐methyl‐2‐pyrrolidone (NMP). And the mass loading of the electrodes was carefully controlled within the range of 0.4–1.0 mg cm^2^. The specific capacity was caculated based on the mass of TiO_2_ within the UNTHS. The resulting slurries were applied to the Cu foil. After heated at 120 °C for 12 h, the sheet was punched into 15 mm diameter electrodes. The cells were assembled in an argon‐filled glove box with the concentrations of moisture and oxygen below 1 ppm. For half‐cell tests, the anode was tested using 2032‐type coin cells, where Li metal was used as as both the counter and reference electrode, and 1.0 M LiPF_6_ in ethylene carbonate/dimethyl carbonate (EC/DMC, 1:1 volume ratio). The cyclic voltammetry and galvanostatic charge–discharge measurements were conducted on a CHI 660B electrochemical workstation (Shanghai CH Instrument Company, China). Cycle‐life measurements for the half‐cell were carried out on a battery test system (Land CT2001A model, Wuhan Land Electronics. Ltd.).

## Supporting information

As a service to our authors and readers, this journal provides supporting information supplied by the authors. Such materials are peer reviewed and may be re‐organized for online delivery, but are not copy‐edited or typeset. Technical support issues arising from supporting information (other than missing files) should be addressed to the authors.

Supplementary
