# Supplementary material for: Self‐Templated Synthesis of Ultrathin Nanosheets Constructed TiO2 Hollow Spheres with High Electrochemical Properties
Source: Adv Sci (Weinh). 2016 Jul 12;3(11):1600162. doi: 10.1002/advs.201600162 (PMC5102664; doi:10.1002/advs.201600162)
Supplement: Supplementary file 1 — Supplementary [file ADVS-3-0n-s001.pdf]

Copyright WILEY-VCH Verlag GmbH & Co. KGaA, 69469 Weinheim, Germany, 2016.

Supporting Information

for *Adv. Sci.*, DOI: 10.1002/advs.201600162

Self-Templated Synthesis of Ultrathin Nanosheets Constructed TiO_2 Hollow Spheres with High Electrochemical Properties

*Huiqi Xie, Linfeng Hu, Feilong Wu, Min Chen, and Limin Wu**

Submitted to ADVANCED SCIENCE Open Access

Copyright WILEY-VCH Verlag GmbH & Co. KGaA, 69469 Weinheim, Germany, 2016.

Supporting Information

Synthesis of Ultrathin Nanosheets Constructed TiO_2 Hollow Spheres with High Electrochemical Properties

*by Huiqi Xie,[+] Linfeng Hu,[+] Min Chen, and Limin Wu**

[*] Prof. L. M. Wu, Dr. H. Q. Hui, Dr. L. F. Hu and Prof. M Chen
Department of Materials Science
Fudan University
Shanghai 200433 (P. R. China)
E-mail: lmw@fudan.edu.cn,

[+] These authors contributed equally to this work.

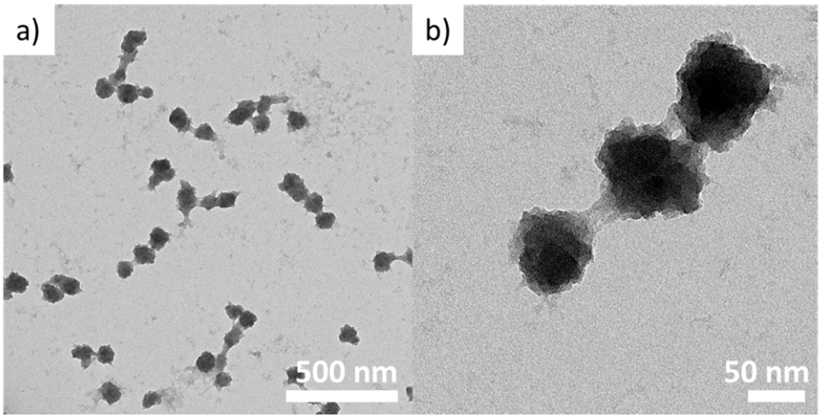

Figure S1. (a, b) Transmisiion electrom microscope (TEM) images of nanoparticles synthesized from TTIP without C_{16}TS olygomers.

Table S1. Atomic ratios of C, O, Si and Ti of the composite particles from EDS results.

**Elements**	**Wt%**	**Atomic %**
C	32.1	49.9
O	29.4	34.2
Si	3.30	2.19
Ti	35.2	13.7
Total	100.0	100.0

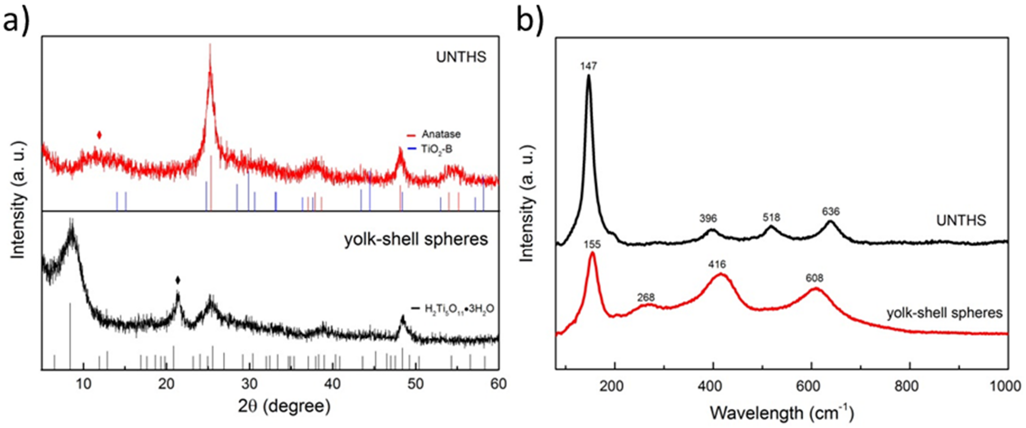

Figure S2. (a) XRD patterns and (b) Raman spectras of the yolk-shell composite spheres and UNTHS.

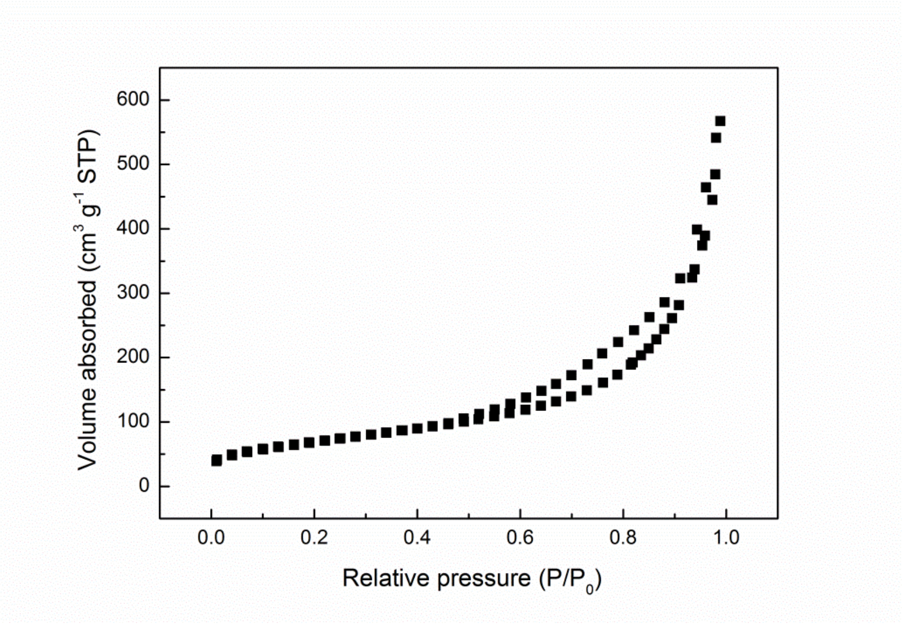

Figure S3. N_2 sorption isotherms of the UNTHS.

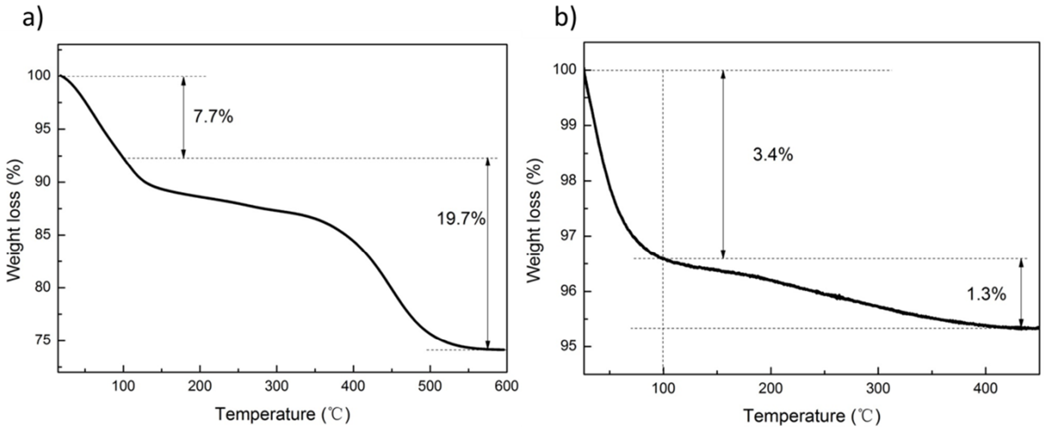

Figure S4. (a) TG-DTG curves of the yolk-shell composite spheres and (b) TG curves of the UNTHS at a heating rate of 10°C/min in flowing dry air gas.

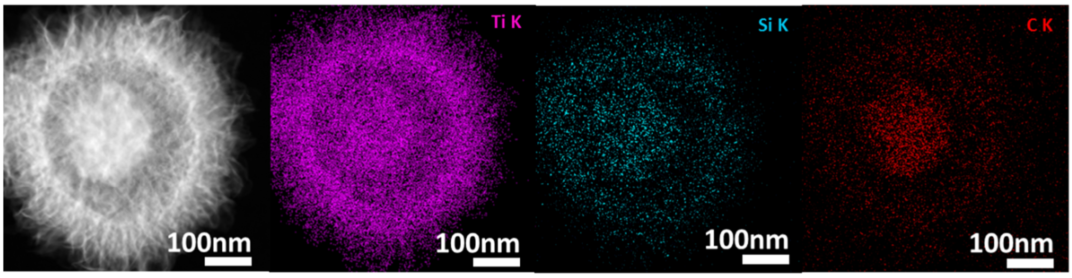

Figure S5. Scanning transmisiion electron microscope (STEM) images of the hydrothermal treated composite particles for 40 min and the EDS mapping images of Ti, C, and Si.

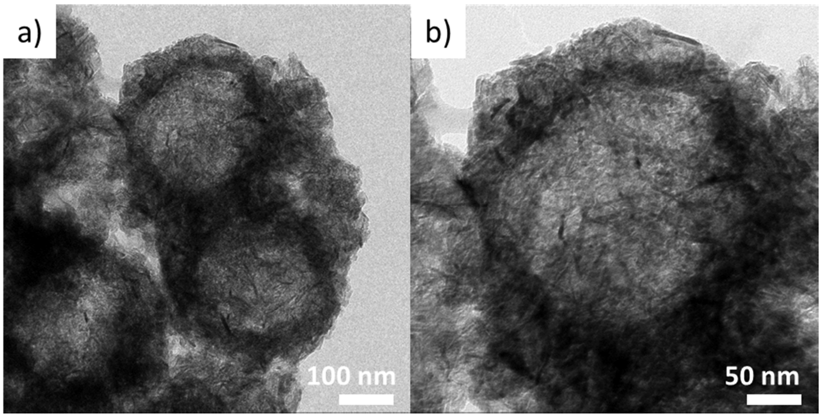

Figure S6. (a, b) TEM images of the UNTHS after 200th charge/discharge cycle.

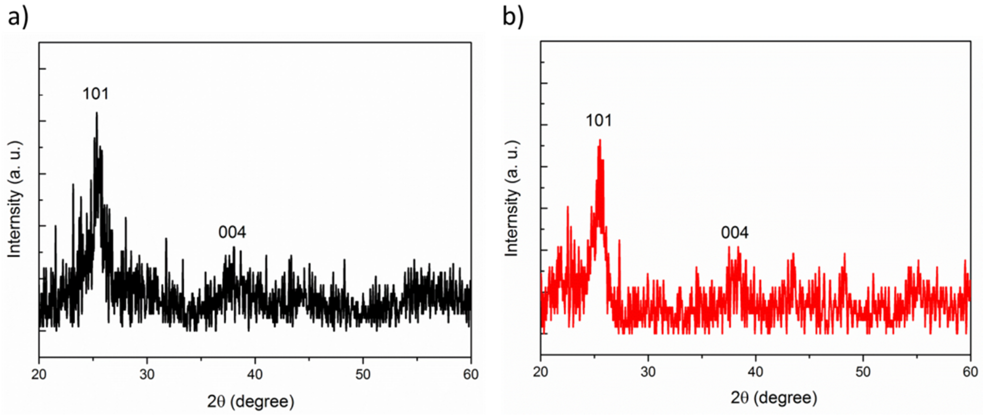

Figure S7. XRD patterns of the UNTHS electrode a) before and b) after 200 charge/discharge cycles at a current density of 20 C.

Table S2. Comparison of electrochemical performance of various TiO_2 based materials.

Electrochemical Performance	Referencess
1C: 151.9 mAh g^{-1}	*J .Am. Chem. Soc*., **2011**, *133*, 933 (Ref. 36)
1 C: discharge capacity 264.8 mA h g^{-1} for the first cycle, 158 mAh g^{-1} after 100 cycles; 10 C: 138 mAh g^{-1}	*Chem. Commun.,* **2010**, *46*, 8252 (Ref. 43)
1 C: 204.1 mA h g-1 after 30 cycles; 20 C: 104.9 mAh g^{-1};	*Chem. Eur. J.* **2016**, *22*, 1 (Ref. 21)
0.5 C: 191 mA h g^{-1}; 1 C: 177 mAh g^{-1}; 25 C: 114 mAh g^{-1}	*J. Am. Chem. Soc.* **2015**, *137*, 13161 (Ref. 37)
1C: 237 mAh g^{-1}; 10 C: 129 mAh g^{-1}	*Nano Lett.* **2014**, *14*, 6679 (Ref. 38)
1C: 147 mAh g^{-1}; 40C: 60.8 mAh g^{-1}	*Angew. Chem. Int. Ed,*. **2014**, *53*, 12590 (Ref. 31)
165 mAh g^{-1} (C rate is not given)	*J. Am. Chem. Soc.*, **2011**, *133*, 933 (Ref.30)
0.5C: 140 mAh g^{-1}; 1C: 110 mAh g^{-1}	*Adv. Mater.* **2012**, *24*, 4124 (Ref. 32)
1C: 132 mAh g^{-1}; 30C: 70 mAh g^{-1}	*Adv. Mater.* **2006**, *18*, 1421 (Ref. 33)
1C: 166 mAh g^{-1}	*Nano Energy* **2014**, *6*, 109 (Ref. 34)
0.5C: 189 mA h g^{-1}; 5C: 116 mAh g^{-1}	*J. Mater. Chem. A,* **2014**, *2*, 2801 (Ref. 35)
0.5 C 210 mAh g^{-1}; 1 C: 200 mAh g^{-1}; 25 C: 100 mAh^{-1}	This work (1.0 V - 3.0 V)
